# Vibrational Characteristics of High-Quality MBE Grown GaAs_1−x−y_Sb_y_N_x_/GaAs (001) Epilayers

**DOI:** 10.3390/ma19050923

**Published:** 2026-02-28

**Authors:** Devki N. Talwar, Hao-Hsiung Lin

**Affiliations:** 1 Department of Physics & Astronomy, University of North Florida, 1 UNF Drive, Jacksonville, FL 32224, USA; 2 Department of Physics, Indiana University of Pennsylvania, 975 Oakland Avenue, 56 Weyandt Hall, Indiana, PA 15705, USA; 3 Department of Electrical Engineering, Graduate Institute of Electronics Engineering, National Taiwan University, Taipei 106319, Taiwan; hhlin@ntu.edu.tw

**Keywords:** dilute III–V-Ns, MBE grown GaAs_1−x−y_Sb_y_N_x_/GaAs (001) epilayers, impurity modes, Green’s function method, rigid-ion-model, Raman spectroscopy

## Abstract

The significant disparity between the size and electronegativity of N and group-V (P, As, Sb) atoms in dilute III–V-Ns remains a cornerstone for developing the next-generation electronics. Variations in the structural, optical, and phonon properties of the quaternary GaAs_1−x−y_Sb_y_N_x_ alloys are being used for improving the high-performance photovoltaic energy and optoelectronic technologies. Bandgap $E_{g}$ tunability has assisted efficient light emission/detection to cover the crucial optical fiber wavelengths for the low-cost integrated chips in data communications and sensing devices. The lattice dynamical properties of these materials are critical for assessing the reliability to evaluate the performance of long-wavelength lasers, photodetectors, and multi-junction solar cells. Our systematic Raman measurements on high-quality MBE grown $Ga{As}_{0.946}{Sb}_{0.032}N_{0.022}$/GaAs samples have detected $\omega_{TO(\Gamma )}^{GaAs}$ and $\omega_{TO(\Gamma )}^{GaAs}$ phonons along with a high frequency N_As_ local mode near ~476 cm^−1^. Weak phonon structures on both sides of the broad 476 cm^−1^ band are interpreted forming a complex N_As_–Ga–Sb_As_ defect center. Using a realistic rigid-ion model in the Green’s function framework, the simulations of impurity modes for isolated and complex defects have provided corroboration to the experimental data.

## 1. Introduction

Comprehending the role of nitrogen (N) in III–V compound semiconductors continue to be a focal point of research in solid-state physics and materials science and engineering [1,2,3,4,5,6,7,8]. Introducing a small amount of N (≤ 5%) in the binary GaAs and ternary Al_x_Ga_1−x_As, In_x_Ga_1−x_As, GaAs_1−x_Sb_x_ alloys results in creating the dilute III–V-Ns. This process substantially modifies the basic properties of conventional III–V compounds, particularly their energy bandgaps $E_{g},$ which allow the fabrication of many optoelectronic devices operating at specific wavelengths. Major devices that have been accomplished using dilute III-nitrides include laser diodes (LDs), photodetectors (PDs) for communication wavelengths (i.e., 1.31–1.55 µm), and high-conversion efficiency multi-junction solar cells (MJSCs), etc.; see refs [9,10,11,12,13,14,15,16]. More recently, these alloys are being considered in developing cost-effective, high-performance modules for nano-/micro-electronics by employing compatible and well-established GaAs fabrication processes [17,18,19,20,21,22,23,24].

Although significant research efforts are made to comprehend the structural, electronic and vibrational properties of GaAs_1−x_N_x_, Al_y_Ga_1−y_As_1−x_N_x_, In_y_Ga_1−y_As_1−x_N_x_ alloys [25,26,27,28,29,30,31,32,33,34,35,36,37,38,39,40,41,42,43,44,45,46,47,48], the quaternary GaAs_1−x−y_Sb_y_N_x_ alloy system has not been explored extensively [24,25,26]. Fundamental properties of this material depend specifically on the microscopic spatial arrangement, and compositions of its constituent elements x, y [29,30,31,32,33,34,35,36,37]. In GaAs_1−x_N_x_, In_y_Ga_1−y_As_1−x_N_x_ and GaAs_1−x−y_Sb_y_N_x_, several N-related defects have been suggested. In addition to an isolated substitutional defect N_As_, N interstitials N_int_, split interstitials (N_int_–N_int_)_As_, (N_int_–As)_As_, nearest neighbor (NN) In_Ga_–N_As_, and next-nearest-neighbor (NNN) N_As_–Sb_As_, N_As_–N_As_ complexes are speculated [24,25,26,29,30,31,32,33,34,35,36,37]. Despite these conjectures, no firm identifications of N-based complex centers are established. Thus, it is highly desirable to adopt experimental and theoretical methods to carefully characterize ternary and quaternary III–V-N alloys. Common studies that have been conducted for evaluating the site-selectivity of N species include the photoluminescence (PL) [48,49], photoreflectance (PR) [10], secondary ion mass spectrometry (SIMS) [49], high resolution X-ray diffraction (HR-XRD) [50], extended X-ray absorption fine structure (EXAFS) [45], nuclear reaction analysis (NRA) [51], electron paramagnetic resonance (EPR) [52], Fourier transform infrared (FTIR) absorption [28,29,30,31,32,33,34,35,36,37], and Raman scattering spectroscopy (RSS) [32,33], etc. HR-XRD is a standard method of determining x in GaAs_1−x_N_x_ that typically relies on Vegard’s law and assumes linear variation in lattice constant $a\left(x\right),\;$with composition [53].

In the conventional semiconductors, both FTIR and RSS have been successfully employed for comprehending the local bonding of isolated and complex defect centers [28,29,30,31,32,33,34,35,36,37]. In doped materials, the observed phonon frequencies and mode degeneracies are used as fingerprints to assess the site symmetry of defects. Vibrational characteristics in dilute ternary GaAs_1−x_N_x_ and quaternary alloys Al_x_Ga_1−x_As_1−y_N_y_ and In_y_Ga_1−y_As_1−x_N_x_ have been reported [28,29,30,31,32,33,34,35,36,37]. In the GaAs_1−x_N_x_ alloy, Wagner et al. [33] noticed broadening of the N_As_ LVM near 471 cm^−1^. Adding In into GaAs_1−x_N_x_ indicated the splitting of the N_As_ mode in Ga_4_N_As_ to three components [33] suggesting the formation of Ga_3_In_1_N_As_. One of the three modes in In_y_Ga_1−y_As_1−x_N_x_ is seen at a frequency higher than that of the N_As_ local mode. This observation indicated strain distribution causing modifications of the local N-bonding. New vibrational features have proposed significant numbers of In_Ga_–N_As_ bonds present in the as-grown state of In_y_Ga_1−y_As_1−x_N_x_ alloys [33]. Very few experimental or theoretical [25,26,44] efforts are made for identifying the N-related vibrational modes in ternary GaAs_1−x_N_x_, GaP_1−x_N_x_, GaSb_1−x_N_x_ and GaAs_1−x−y_Sb_y_N_x_ quaternary alloys.

Two approaches are frequently used to study the lattice dynamics of perfect/imperfect semiconductors. These are (i) the microscopic or first-principles methods [44,54,55,56,57,58], which start with an ionic potential screened by electron gas for deriving the structural and vibrational properties, and (ii) the macroscopic techniques which employ phenomenological models [59,60,61,62,63,64,65,66,67,68,69] in terms of the general interatomic forces. Earlier, Buckeridge et al. [44] reported N-related LVMs in dilute ternary GaAs_1−x_N_x_ and GaSb_1−x_N_x_ alloys by adopting a density functional method (DFT). For studying the dynamical properties of impurities, many researchers have preferred macroscopic methods using Green’s function (GF) formalism [59,60,61,62,63,64,65,66,67,68,69]. Careful analysis of LVMs for donors and acceptors in semiconductors has achieved important revelations [65]. Estimated changes in the nearest-neighbor (NN) impurity–host interactions are ascribed to the electric field created (cf. Section 3.2) by charged impurities [65,66]. This important fact is used here to study the LVMs of isoelectronic $N_{As(Sb)}$, acceptors $C_{As(Sb)}$, and $N_{As(Sb)}$–$N_{As(Sb)}$ pair defects in GaAs_1−x_N_x_ and GaSb_1−x_N_x_ alloys. The effects of adding In and Sb in GaAs_1−x_N_x_ alloys are meticulously examined by the GF method [24,25,26] assuming the formation of NN $N_{As}-{In}_{Ga}$ and the NNN $N_{As}-Ga-{Sb}_{As}$ complexes.

Simulations of impurity vibrational modes by GF theory require accurate values of the phonon dispersions $\omega_{j}(\vec{q})$ of perfect crystals. To calculate $\omega_{j}(\vec{q})$ of GaAs and GaSb, we have adopted a realistic rigid-ion model (RIM) [59]. Eigen values and eigenvectors of the host crystals are carefully integrated to obtain the GF matrix elements (${\overset{⃡}{G}}^{o}$). Appropriate perturbation matrices ($\overset{⃡}{P}$) are methodically constructed by including mass changes at the impurity sites as well as NN interactions between the impurity and host lattice atoms for isolated and complex defect centers. The comparison of GF calculations with the DFT [44] study and existing Raman scattering and/or FTIR measurements [28,29,30,31,32,33,34,35,36,37] has helped us in identifying the nature of different types of defect centers in dilute GaAs_1−x_N_x_, GaSb_1−x_N_x_ and GaAs_1−x−y_Sb_y_N_x_ alloys.

This paper aims to report the results of systematic experimental and theoretical studies on the MBE grown GaAs_1−x−y_Sb_y_N_x_/GaAs (001) epilayers (cf. Section 2.1, Section 2.2 and Section 2.3). A set of $Ga{As}_{0.946}{Sb}_{0.032}N_{0.022}$ samples are prepared with the low composition of N and Sb. GaAs, GaN, and GaSb are used as standards for the ZAF (i.e., atomic number Z, absorption A, and fluorescence F) correction. Thermal annealing of the samples is carried out in a N_2_ gas environment at 850 °C for 5 min. The annealing temperature is calibrated by monitoring the melting of Al films coated on Si wafers. Room temperature (RT) Raman spectra are recorded in the backscattering geometry by using a Renishaw in Via micro-Raman system with a 1800 grooves/mm grating and optical microscope with a 50 × objective lens. In these measurements a 514.5 nm excitation line of an Ar-ion laser is used with a power of about 1.5 mW by focusing the beam onto a spot size of 5 μm diameter. Raman measurement has indicated a high frequency N_As_ local $(\omega_{loc})\;$mode close to ~476 cm^−1^. In addition to the two-phonon features of optical $\omega_{LO}^{GaAs}\;$and $\omega_{TO}^{GaAs}$ modes for GaAs, the study has also revealed weak phonon structures on both sides of the N_As_ impurity mode. In dilute In_y_Ga_1−y_As_1−x_N_x_ and GaAs_1−x−y_Sb_y_N_x_ alloys (see Section 3), we have described the possible structures of N_As_ ($T_{d}$), N_As_–In_Ga_ ($C_{3v}$), and N_As_–Ga–Sb_As_ ($C_{s}$) defects with appropriate point group symmetries. A realistic RIM [59] is adopted (cf. Section 3.1) for calculating $\omega_{j}(\vec{q})$ of GaAs and GaSb materials. The simulated results (cf. Section 4.1) of $\omega_{j}(\;\vec{q})$ and density of states g(ω) are compared with the inelastic neutron scattering (INS) [70,71] and first-principles calculations [44,56]. Green’s functions ${\overset{⃡}{G}}^{o}$(ω) of the perfect materials and perturbation $\overset{⃡}{P}$(ω) matrices are required to study the impurity vibrational modes of different defect centers (cf. Section 4.2 and Section 4.3). In describing $\overset{⃡}{P}$, we did not include the changes in the Coulomb forces, as their long-range interactions would render the GF method intractable. Calculations of the LVMs are performed to assess the phonon features of various defect centers in the GaAs and GaSb materials. Theoretical calculations are compared and discussed (cf. Section 4) with the experimental [28,29,30,31,32,33,34,35,36,37] and DFT methods [44]. Conclusions are drawn in Section 5.

## 2. Experimental Procedure

$Ga{As}_{0.946}{Sb}_{0.032}N_{0.022}$/GaAs (001) epilayers are prepared (cf. Section 2.1). Post-growth rapid thermal annealing (RTA) is carried out at 850 °C (cf. Section 2.2). Room temperature Raman measurements (cf. Section 2.3) revealed a N_As_ LVM near ~476 cm^−1^. Large full width at half maximum (FWHM) with weak features on both sides of 476 cm^−1^ suggest a strong affinity of N_As_ and Sb_As_ with favorable bonding to create a N_As_–Ga–Sb_As_ NNN pair.

### 2.1. MBE Growth of GaAs_1−x−y_Sb_y_N_x_/GaAs

By using a VG-V80H gas source MBE system, USA we have grown high-quality 1 μm thick layers of $Ga{As}_{0.946}{Sb}_{0.032}N_{0.022}$ on semi-insulating GaAs (001) substrates. Gas sources are used for the elements like As and N along with a high purity EPI SUMO cell for the Ga to ensure minimal impurities. Pure arsine (AsH_3_) precursor is injected into the gas cell and cracked at 1000 °C to provide the As_2_ beam. The Sb_4_ flux from the reservoir zone is cracked at 1050 °C to supply a mixed beam of Sb_2_ dimer and Sb monomer. The N source is generated by injecting ultrahigh purity N_2_ gas to an EPI Uni-bulb RF plasma cell.

### 2.2. Rapid Thermal Annealing

Rapid thermal annealing is a critical post-growth step to help improve the material quality. A tabletop ULVAC MILA-3000 RTA vacuum system, USA is used for high temperature testing and analysis of $Ga{As}_{0.946}{Sb}_{0.032}N_{0.022}$/GaAs (001) samples. The system is equipped with an infrared gold image furnace, a quartz glass chamber along with a programmable digital T controller. It offers high-speed heating with precise control of T up to 1200 °C in less than 24 s. To avoid oxidation, the chamber is filled with N_2_ ambient gas during thermal treatment. Before carrying out the RTA process, the samples are protected by a blank Si substrate. This acts as a physical barrier which maintains a localized overpressure of the species and significantly reduces the material loss. Temperature is calibrated by a Si substrate overlaid with an Al layer that relies on Si-Al eutectic point to ensure process congruity. Thermal annealing is carried out in N_2_ ambient gas at 850 °C for 5 min.

### 2.3. Raman Spectroscopy

On $Ga{As}_{0.946}{Sb}_{0.032}N_{0.022}$/GaAs (001) epilayers, RT Raman measurements are performed in the backscattering geometry. A Renishaw in Via micro-Raman system is used with a 514.5 nm excitation line of the Ar-ion laser. In this configuration, the incident and scattered light beams are parallel to the [001] direction of the epilayer. One expects perceiving only a $\omega_{LO(\Gamma )}^{GaAs}\;$mode as the $\omega_{TO(\Gamma )}^{GaAs}\;$phonon is forbidden by symmetry-based Raman selection rules [32,33,34,35].

In $Ga{As}_{0.946}{Sb}_{0.032}N_{0.022}$ alloy, not only have we observed $\omega_{TO(\Gamma )}^{GaAs}$ mode but also two-phonon features of GaAs along with a high frequency N_As_ LVM near 476 cm^−1^ of $T_{d}$ symmetry appearing in the broad band region with a larger FWHM (cf. Section 4.2). This has suggested the possibility of N_As_ and Sb_As_ atoms forming a localized pair, specifically the N_As_–Ga–Sb_As_ complex center of reduced $C_{s}$ symmetry.

## 3. Theoretical Background

Dilute III–V-Ns are prototypical materials with an extreme disparity between the size and electronegativity of N and group-V anions. This mismatch has caused many unique structural, electronic and phonon characteristics [25,26,27,28,29,30,31,32,33,34,35,36,37,38,39,40,41,42,43,44,45,46]. Adding a small amount of group-III (Al, In) cations or group-V (Sb) anions in GaAs_1−x_N_x_ has created Al_y_Ga_1−y_As_1−x_N_x_, In_y_Ga_1−y_As_1−x_N_x_ or $Ga{As}_{1-x-y}{Sb}_{y}N_{x}\;$quaternary alloys. Many studies [25,26,27,28,29,30,31,32,33,34,35,36,37,38,39,40,41,42,43,44,45,46] have proposed preferential bonding between N_As_ and Al_Ga_, In_Ga_, or Sb_As_ atoms. Spectroscopic measurements are reported showing specific phonon features. Limited simulations [44] exist to confirm the formation of such favored defect centers. Here, we have studied the impurity vibrational modes (cf. Section 3.2) of isolated, pair, and complex defect centers using a GF methodology [63,64,65,66] in the framework of an RIM [59].

### 3.1. Rigid-Ion Model

The RIM proposed by Kunc [59] is succinctly outlined here to comprehend the INS data of GaAs and GaSb [70,71]. The quantities of interest in this model are the force constants$\;{\overset{⃡}{Ф}}^{sC}\left[\equiv {\overset{⃡}{Ф}}^{s}+{\overset{⃡}{Ф}}^{C}\right]\;$or dynamical ${\overset{⃡}{D}}^{sC}\left[\equiv {\overset{⃡}{D}}^{s}+{\overset{⃡}{D}}^{C}\right]\;$matrices. In zinc blende (zb) materials, it includes short-range interactions (${\overset{⃡}{Ф}}^{s}$) up to second NN (A, B,$C_{\kappa }$,$D_{\kappa }$,$E_{\kappa }\;$and $F_{\kappa }$; κ = 1, 2) and long-range Coulomb interactions $({\overset{⃡}{Ф}}^{C\;})\;$using an effective charge parameter $Z_{eff}(\equiv Z_{\kappa }e)$. In harmonic approximation, the lattice vibrations $\omega_{j}(\vec{q})$ are obtained by solving [59]

(1) $$\omega_{j}^{2}(\vec{q})e_{\alpha }(\kappa |\vec{q}j)=\sum_{\kappa^{\prime }\beta }D_{\alpha \beta }^{sC}(\kappa \kappa^{\prime }|\vec{q})e_{\beta }(\kappa^{\prime }|\vec{q}j);\kappa ,\kappa^{\prime }=1,2,$$

where $D_{\alpha \beta }^{sC}(\kappa \kappa^{\prime }|\vec{q})\left[\equiv D_{\alpha \beta }^{s}(\kappa \kappa^{\prime }|\vec{q})+D_{\alpha \beta }^{C}(\kappa \kappa^{\prime }|\vec{q})\right]\;$represents the dynamical matrix comprising the short- $D_{\alpha \beta }^{s}(\kappa \kappa^{\prime }|\vec{q}),$ and long-range Coulomb $D_{\alpha \beta }^{C}(\kappa \kappa^{\prime }|\vec{q})$ interactions.

For each mode, the eigenvectors $e_{\alpha }(\kappa |\vec{q}j)$ in Equation (1) satisfy the familiar orthogonality

(2a) $$\sum_{\alpha \kappa }e_{\alpha }^{*}(\kappa |\vec{q}j)e_{\alpha }(\kappa |{\vec{q}j}^{\prime })=\delta_{jj^{\prime }},$$

and closure relations

(2b) $$\sum_{j}e_{\alpha }^{*}(\kappa^{\prime }|\vec{q}j)e_{\beta }(\kappa |{\vec{q}j}^{\prime })=\delta_{\kappa \kappa^{\prime }}\delta_{\alpha \beta }.$$

Once the interatomic force constants (IFCs) $[A,B,C_{\kappa },D_{\kappa },E_{\kappa },F_{\kappa }$, and $Z_{eff}$] are evaluated for GaAs and GaSb [59] (cf. Section 4.1), it is straight forward to simulate $\omega_{j}(\vec{q})$.

### 3.2. The Green’s Function Approach

In the GF methodology, by considering appropriate symmetries of various defect centers [72], it is possible to evaluate LVMs by visualizing and identifying modes which are optically active and remain localized around the defects. A detailed account of the dynamical properties of perfect/imperfect crystals by the GF method has been reported [63,64,65,66] elsewhere. Our discussion to treat the impurity vibrational modes in the GaAs and GaSb is very brief and only for the purpose of establishing important notations to be used throughout the paper.

#### 3.2.1. The Perfect Lattice Green’s Functions

The perfect lattice GF, ${\overset{⃡}{G}}^{o},$ can be expressed in the matrix notation as [63,64,65,66]

(3) $$(\overset{⃡}{M}\omega^{2}-{\overset{⃡}{Ф}}^{sC})\;{\overset{⃡}{G}}^{o}=\overset{⃡}{I},$$

where the eigenfrequencies of the host crystal are obtained by solving the equation:

(4) $$\det\;[(\overset{⃡}{I}\omega^{2}-{\overset{⃡}{D}}^{sC})]\;=\;\det{\left[{\overset{⃡}{G}}^{o}\left(\omega \right)\right]}^{-1}/\det\left[\overset{⃡}{M}\right],$$

The component form of ${\overset{⃡}{G}}^{o}$ matrix can be defined as

(5) $$<l\kappa \left|G_{\alpha \beta }^{o}\left(\omega \right)\right|l^{\prime }\kappa^{\prime }>=\frac{1}{ℵ{\left(M_{\kappa }M_{\kappa^{\prime }}\right)}^{1/2}}\sum_{\vec{q}j}\frac{e_{\alpha }(\kappa |\vec{q}j)e_{\beta }^{*}(\kappa^{\prime }|\vec{q}j)}{{(\omega +i0}^{+}{)}^{2}-\omega_{j}^{2}(\vec{q})}\times exp\left\{i\vec{q}[\vec{x}\left(l\kappa \right)-\vec{x}\left(l^{\prime }\kappa^{\prime }\right)]\right\}.$$

Here, ℵ denotes the number of wave vectors and $\vec{x}\left(l\kappa \right)$ is the equilibrium position vector of an atom (lκ). An infinitesimal positive imaginary value to ω is added for producing the retarded GF with a sinusoidal time dependence. The elements of ${\overset{⃡}{G}}^{o}$ (Equation (5)) can be expressed in terms of its real and imaginary parts [63,64,65,66]. The real part of the matrix $<l\kappa \left|Re\;G_{\alpha \beta }^{o}\left(\omega \right)\right|l^{\prime }\kappa^{\prime }>\;$is the principal segment of Equation (5), while the imaginary part $<l\kappa \left|Im\;G_{\alpha \beta }^{o}\left(\omega \right)\right|l^{\prime }\kappa^{\prime }>$ can be obtained by using

(6) $$<l\kappa \left|Im\;G_{\alpha \beta }^{o}\left(\omega \right)\right|l^{\prime }\kappa^{\prime }>=\frac{\pi }{ℵ{\left(M_{\kappa }M_{\kappa^{\prime }}\right)}^{\frac{1}{2}}}\sum_{\vec{q}j}e_{\alpha }\left(\kappa |\vec{q}j\right)e_{\beta }^{*}\left(\kappa^{\prime }|\vec{q}j\right)\times exp\left\{i\vec{q}[\vec{x}\left(l\kappa \right)-\vec{x}\left(l^{\prime }\kappa^{\prime }\right)]\right\}\times \delta (\omega^{2}-\omega_{j}^{2}(\vec{q})),$$

Clearly, Equation (6) becomes zero outside the range of the allowed phonon frequencies of the host crystal lattice. For numerical calculations of GFs, we have followed standard procedures by obtaining first the imaginary part from a sample of wave vectors $\vec{q}$ in the reduced Brillouin zone (BZ) and then determining the real part via the links provided by Kramers–Krönig relations [63,64,65,66].

#### 3.2.2. The Imperfect Lattice Green’s Functions

Like ${\overset{⃡}{G}}^{o}\;$of the perfect lattice, one can write GF matrix elements for the imperfect crystal $\overset{⃡}{G}$ by using

(7) $$[(\overset{⃡}{M}+∆\overset{⃡}{M})\omega^{2}-({\overset{⃡}{Ф}}^{sC}+∆{\overset{⃡}{Ф}}^{sC})]\;\overset{⃡}{G}=\overset{⃡}{I},$$

or equivalently in the form of a Dyson’s equation:

(8) $$\overset{⃡}{G}\left(\omega \right)=[\overset{⃡}{I}-{\overset{⃡}{G}}^{o}(\omega )\;\overset{⃡}{P}(\omega ){]}^{-1}{\overset{⃡}{G}}^{o}(\omega ).$$

In Equation (8), the term $\overset{⃡}{P}\left(\omega \right)[\equiv -∆\overset{⃡}{M}\omega^{2}+∆{\overset{⃡}{Ф}}^{sC}]$ is the perturbation matrix caused by defects. The quantities $∆\overset{⃡}{M}\;$and$\;∆{\overset{⃡}{Ф}}^{sC}[\equiv ∆{\overset{⃡}{Ф}}^{s}+\;∆{\overset{⃡}{Ф}}^{C}]$ represent the mass and force constant change matrices. Since the variation in Coulomb interactions is set to zero $∆{\overset{⃡}{Ф}}^{C}\;$= 0, we will consider only the mass change at the impurity sites and variation in the NN impurity–host interactions (cf. Section 3.2.3) in defining $\overset{⃡}{P}\;$for different defect centers. The impurity vibrational modes can be obtained by solving the equation [63,64,65,66]:

(9) $$det|[\overset{⃡}{I}-{\overset{⃡}{G}}^{o}(\omega )\;\overset{⃡}{P}\left(\omega \right)]|=0.$$

Equation (9) provides the poles of $\overset{⃡}{G}\left(\omega \right)$: (i) as an LVM at energy above the maximum phonon frequency of the bulk material, (ii) a gap mode (GM) in the space between acoustic and optic modes and, (iii) an in-band mode falling within the host lattice phonons [63,64,65,66]. To simulate the impurity modes of various defect centers, we took advantage (cf. Section 3.2.4) of the symmetry-adapted algorithm [72].

#### 3.2.3. Perturbation Matrices

**Figure 1:**
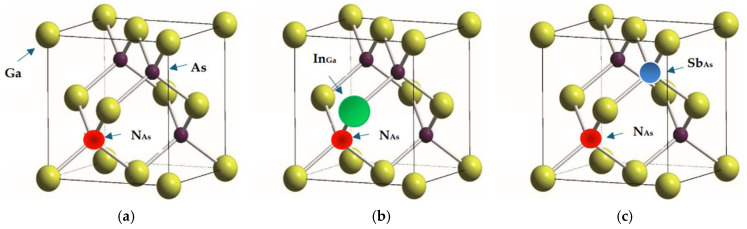
Perturbation models for defects in the zinc blende GaAs. (**a**) An isolated defect N_As_ of $T_{d}$ symmetry: N atom is shown by red color solid sphere occupying the As site κ = 2. (**b**) Nearest-neighbor pair defect N_As_–In_Ga_ of $C_{3v}$ symmetry: N_As_ and In_Ga_ (green color solid sphere) are occupying κ = 2 and κ = 1 sites, respectively. (**c**) Next-nearest-neighbor complex centers N_As_–Ga–Sb_As_ ${of\;C}_{s}$ and N_As_–Ga–N_As_ $of\;C_{2v}$ symmetry: N_As_ on As-site and N_sb_ (blue color solid sphere) occupying the 2nd nearest As site. If the two impurities are identical the symmetry is $C_{2v};$ otherwise $C_{s}$ (see: text).

In any defect calculations, the most important issue is to give an adequate representation of the impurity perturbation $\overset{⃡}{P}$. To study the dynamical behavior of defects (see Figure 1) using the GF method, we have appropriately constructed $\overset{⃡}{P}(\omega )$ by considering the effects of lattice relaxation to account for the impurity–host interactions. Lattice relaxation in the vicinity of substitutional impurities is estimated using Harrison’s semiempirical bond-orbital model [73]. In terms of Hartee–Fock atomic term values, this method provides simple analytical expressions for the change in impurity–host and host–host bond energies and suggests a computationally efficient and reasonably accurate way of estimating the bond-length distortions. In the framework of RIM, the perturbation matrices $\overset{⃡}{P}(\omega )$ are constructed following the method described in ref. [63]. To obtain $\overset{⃡}{P}(\omega )$ we have used the scaling properties of lattice relaxation caused by different isolated defects along with the trends of short-range interactions in different II–VI and III–V host crystals.

#### 3.2.4. Defect Symmetry Considerations

(a) Isolated defects: $T_{d}$ symmetry

In GaAs the simplest defect responsible for impurity vibrational modes is an isolated substitutional impurity of $T_{d}\;$symmetry (see Figure 1a,b), where the host lattice atom As (κ = 2) or Ga (κ = 1) [63] is replaced by iso-electronic $N_{As}\;$or ${In}_{Ga}$ atoms, respectively. In the framework of RIM, the perturbation matrix $\overset{⃡}{P}(\omega )$ includes both the changes in atomic masses at impurity sites and the NN force constants (cf. Section 3.1). These changes are expressed by the following parameters:

(10a) $$\varepsilon_{2}=\;(M_{2}-\;M_{2}^{imp})/M_{2},$$

(10b) $$u=(A-A^{\prime \prime })/A=(B-B^{\prime \prime })/B=1-b,$$

or

(10c) $$\varepsilon_{1}=(M_{1}\;-M_{1}^{imp})/M_{1},$$

(10d) $$t=(A-A^{\prime })/A=(B-B^{\prime })/B=1-a,$$

with the impurity of mass $M_{2}^{imp}$ or $M_{1}^{imp}$ occupying either the site κ = 2 or 1, respectively. Following Vandevyver and Plumelle [63], we have considered the impurity–host interaction by a single dimensionless parameter u or t. The stipulation of a A = a B in Equation (10b,d) for delineating the $\overset{⃡}{P}(\omega )$ matrix hardly affects the high frequency LVMs. However, imposing this condition on $\overset{⃡}{P}(\omega )$ satisfies the rotational invariance requirement, which is explicitly invariant with respect to the translations and crystal-symmetry operations [74].

The constructions of 15 × 15 full-size ${\overset{⃡}{G}}^{o}(\omega )$ and $\overset{⃡}{P}(\omega )$ matrices are reported in ref. [63]. Considering the $T_{d}\;$symmetry, we have decomposed ${\overset{⃡}{G}}^{o}(\omega )$ and $\overset{⃡}{P}(\omega )$ into blocks corresponding to the irreducible representations of the group [74]:

(11) $${\Gamma_{T}}_{d}=\;A_{1}⊗\;E\;⊗\;F_{1}⊗\;3F_{2}.$$

The frequencies of local, gap or in-band modes can be obtained in different irreducible representations by solving the real part of the determinant [63]:

(12) $$\prod_{\mu \Gamma }det|[\overset{⃡}{I}-{\overset{⃡}{G}}_{\mu \Gamma }^{o}\left(\omega \right)\;{\overset{⃡}{P}}_{\mu \Gamma }\left(\omega \right)]|=0$$

Here, the ${\overset{⃡}{G}}_{\mu \Gamma }^{o}$ (ω) of perfect lattice GF is projected onto the defect space, and ${\overset{⃡}{P}}_{\mu \Gamma }\left(\omega \right)$ is the perturbation matrix in each ($A_{1}$, E, $F_{1}$, and $F_{2}$) irreducible representation. One must note that the impurity vibrational modes in $A_{1}$, E, and $F_{2}$ representations are Raman active, while the triply degenerate $F_{2}$ mode is IR and Raman active [75,76].

(b) NN Pair Defects: $C_{3v}$ Symmetry

The perturbation matrix for an NN pair defect in zb GaAs involves two impurity atoms occupying the sites 2 and 1 (cf. Figure 1b), respectively, causing changes in masses at impurity sites, i.e., $\varepsilon_{2}$= ($M_{2}$−$M_{2}^{imp}$)/$M_{2}$, $\varepsilon_{1}$= ($M_{1}$−$M_{1}^{imp}$)/$M_{1},$ and force constants between impurity–host atoms, i.e., u and t. An effective force constant between impurities $F_{12}$ (≡ 1 − ab + $\Gamma_{12}$ = u + t − ut + $\Gamma_{12}$) is included (see ref. [64] using $\Gamma_{12}$) to account for the changes in u and t of isolated impurities involved in the formation of a pair defect. The term $F_{12}$ < 0 (or > 0) signifies stiffening (or softening) between the pair bonds. The pair defect of point group symmetry $C_{3v}$ involves eight atoms which cause the size of the impurity space to increase to 24 × 24. The total representation of $C_{3v}$ in the 24-dimensional space group reported by Ludwig [74] is used to block-diagonalize ${\overset{⃡}{G}}^{o}(\omega )$ and $\overset{⃡}{P}(\omega )$ matrices with each block along the diagonal belonging to the following irreducible representations:

(13) $${\Gamma_{C}}_{3v}=\;{6A}_{1}⊗\;{2A}_{2}⊗8\;E.$$

From group theoretic analysis, it is perceived that in the $A_{2}$ representation the impurity atoms remain stationary. Thus, only $A_{1}$ and E type modes are optically active. As the degeneracies of $F_{2}$ mode are lifted at each defect site, one expects observing four LVMs for a pair defect with very light impurity atoms: two non-degenerate modes due to the movement of impurity atoms along the bond [i.e., $\omega_{1}$ ($A_{1}^{+}$ ← →) and $\omega_{4}$ ($A_{1}^{-}$ → →)] and two doubly degenerate modes as a result of their vibration perpendicular to it [i.e., $\omega_{2}$ ($E^{+}$↑↓) and $\omega_{3}$ ($E^{-}$↑↑)], generally with $\omega_{1}$ > $\omega_{2}$ > $\omega_{3}$ > $\omega_{4}$ (cf. Section 4.3). On the other hand, only two ($A_{1}$, E) impurity modes will appear in a pair defect involving a vacancy (or heavy atom) and a light impurity atom. We will use this model to account for the impurity modes of the NN pair defect ($N_{As}-{In}_{Ga}$) in In_y_Ga_1−y_As_1−x_N_x_ [25,26,27,28,29,30,31,32,33,34,35,36,37,38,39,40,41,42,43,44,45,46].

(c) Complex Defects: $C_{s}$ or $C_{2v}\;$ Symmetry

The method used for NN pair defects can be extended to define the perturbation matrix $\overset{⃡}{P}\left(\omega \right)$ for a complex center comprising two substitutional impurities (see Figure 1c) occupying the $N_{As}$ at site (2) and $N_{Sb}$ at the second NN site, respectively. Following the $C_{3v}$ case we have considered the mass change parameter at $N_{Sb}$ in terms of $\varepsilon_{6}$ = ($M_{2}$−$M_{6}^{imp}$)/$M_{2}$ and the force constant variation between impurity–host bonds by v = (A − $A^{\prime \prime \prime }$)/A = (B − $B^{\prime \prime \prime }$)/B = 1 − c. Like the NN pair defect, an effective force constant between the impurity–host atoms 2–6 (≡$F_{26}$) and 1–2 (≡$F_{12}$) is also included. The point group symmetry for such a complex defect center is $C_{2v}$ if $\varepsilon_{2}$ = $\varepsilon_{6}$, and otherwise $C_{s}.$ This complex causes the size of the defect space to increase to 33 × 33. By constructing a total representation of $C_{2v}$/$C_{s}$ in the 33-dimensional space, we have block-diagonalized ${\overset{⃡}{G}}^{o}(\omega )$ and $\overset{⃡}{P}(\omega )$ matrices belonging to the following irreducible representations [74]:

(14a) $${\Gamma_{C}}_{2v}=\;{10A}_{1}⊗{6A}_{2}⊗{8B}_{1}⊗\;{9B}_{2},$$

and

(14b) $${\Gamma_{C}}_{s}={19A}_{1}⊗{14A}_{2},$$

with $A_{1}$, $B_{1}$, and $B_{2}$ ($A_{1}$ and $A_{2}$) types of vibrations being optically active. This perturbation model is used to account for the experimental results on impurity modes of NNN pair defects (e.g., $N_{As}-Ga-{Sb}_{As}$; $N_{As}-Ga-N_{As}$) for analyzing the atypical Raman scattering data (see Section 4) in $Ga{As}_{1-x-y}{Sb}_{y}N_{x}$.

## 4. Numerical Computation Results and Discussions

Dilute III–V-Ns are a new class of alloys for optoelectronic and photovoltaic applications [1,2,3,4,5,6,7,8,9]. $Ga{As}_{1-x-y}{Sb}_{y}N_{x}\;$belongs to this family of materials. Lattice dynamics plays an important role in designing cost-effective, high-performance modules required in nano-/micro-electronics for strain management.

### 4.1. Phonon Properties of GaAs and GaSb

**Figure 2:**
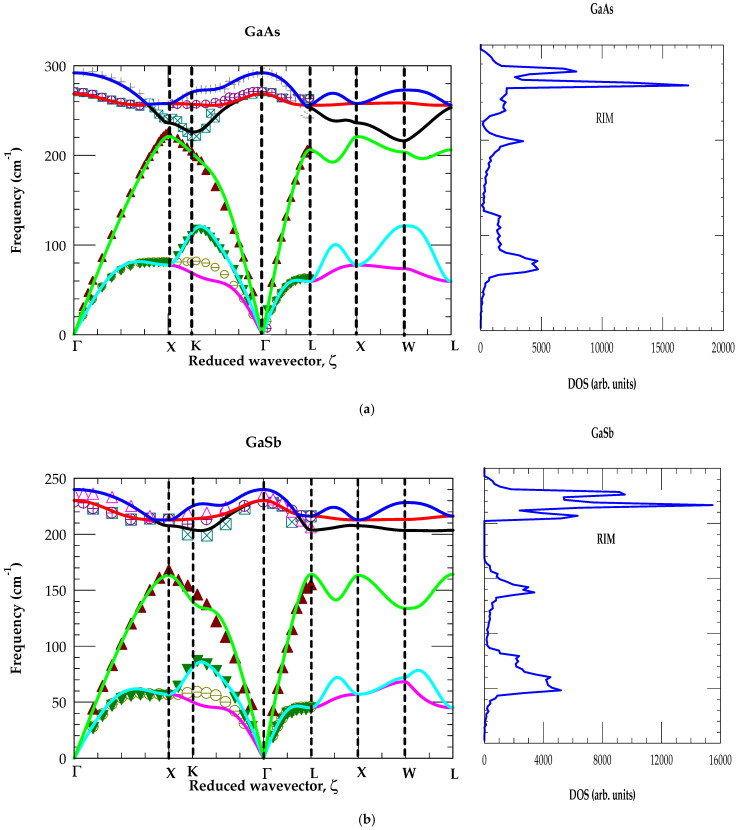
Calculated phonon dispersions (**left** panel) and one phonon density of states (**right** panel) of GaAs (**a**) and GaSb (**b**). Phonon values are compared with INS data [70] of GaAs and GaSb [71].

The best fit inter-atomic force constants for the bulk GaAs and GaSb materials are listed in Table 1. Using these parameters the phonon dispersions $\omega_{j}(\vec{q})$ and one phonon density of states g(ω) are reported in Figure 2a,b).

The results are compared reasonably well with the existing INS [70,71] and Raman scattering measurements. Discrepancies between the calculated and experimental optical and acoustic phonon frequencies are less than 5%. The phonon gap in GaSb falls between 166 and 202 cm^−1^. It is worth stating that the set of IFCs providing good agreement to INS and/or RSS data for perfect crystals is not necessarily a guarantee for the accuracy of a model. The point that compels recognition to a lattice dynamical approach demands simultaneously the correct values of eigenvalues and eigenvectors. Despite the impetus to examine the RIM comes from ab initio methods [44,56], the later approaches have not yet replaced the former schemes completely. The simple reason is that the ab initio methods deal only with phonons at a few high symmetry points while the efficiency of phenomenological models comes to profit when different BZ averages are evaluated in calculating the GFs for studying the impurity vibrations.

Our investigation of LVMs [63,64,65,66] identifying the microscopic lattice structures in GaAs has provided indirect support for the reliability of calculated phonons by the RIM [59]. We will use this methodology to investigate impurity vibrational features observed in Raman scattering studies of $Ga{As}_{1-x-y}{Sb}_{y}N_{x}$ alloys (cf. Section 4.2).

### 4.2. Raman Scattering of $Ga{As}_{0.946}{Sb}_{0.03}N_{0.022}$

**Figure 3:**
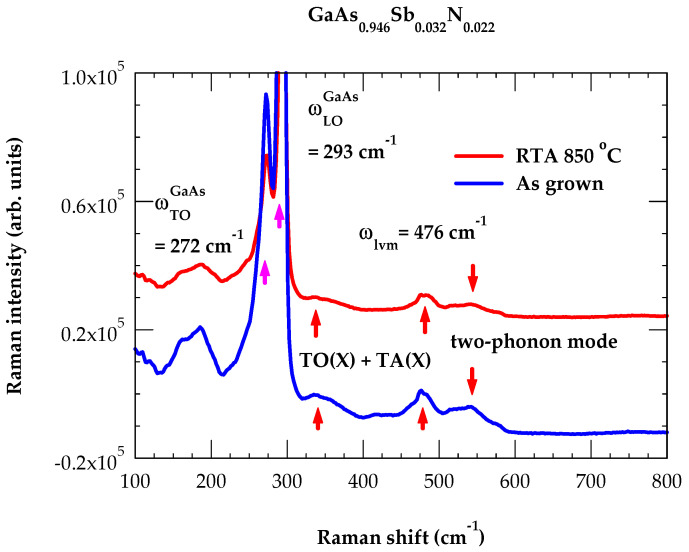
Raman spectra of as-grown and 850 °C annealed $Ga{As}_{0.946}{Sb}_{0.032}N_{0.022}/GaAs\;$ prepared by MBE. These measurements are performed at room temperature in the backscattering geometry. In addition to observing structures near the high frequency LVM of N_As_, we noticed several phonon features of GaAs. Our spectral results are in good agreement with those reported by Milanova et al. [26] on GaAsSb:N bulk layers grown by liquid phase epitaxy.

For the as-grown (AG) and 850 °C annealed $Ga{As}_{0.946}{Sb}_{0.032}N_{0.022}/GaAs$(100) sample, we have reported results of our Raman scattering measurement in Figure 3. Obviously, it has revealed many features occurring at frequencies lower and higher (i.e., $\omega_{m}^{GaAs}>\omega >\omega_{m}^{GaAs})\;$than the maximum phonon frequency of GaAs (shown by different color vertical arrows). Two main modes $\omega_{LO(\Gamma )}^{GaAs}$ and $\omega_{TO(\Gamma )}^{GaAs}$ at 293 cm^−1^ and 276 cm^−1^ (magenta color arrows) exhibit almost identical phonon values with the bulk GaAs [70]. This suggests that in the $Ga{As}_{0.946}{Sb}_{0.032}N_{0.022}\;$sample (with low Sb and N concentration) the vibrational properties are dominated by a strong long-range Ga-As bond network rather than the impurity atoms. The observation of$\;\omega_{TO(\Gamma )}^{GaAs}\;$mode is due to the breakdown of a standard selection rule and possibly caused by disorder. A feature between 150 and 200 cm^−1^ is ascribed to the zone boundary $\omega_{LA(L)}^{GaAs}$ phonon. Our study has also recognized two-phonon features (red color arrows) in the frequency range of 300–350 cm^−1^ and 500–550 cm^−1^. In $Ga{As}_{0.946}{Sb}_{0.032}N_{0.022}$, the LVM of $N_{As}$476 cm^−1^ appears (cf. Figure 3) at a slightly higher frequency than in Ga*As*:N. It falls, however, in a broad band region of 450–500 cm^−1^ with weak features on both sides of 476 cm^−1^. Similar results are reported earlier in AG and annealed GaAsSbN samples [25,26]. It is possible that N_As_ and Sb_As_ atoms form a N_As_–Ga–Sb_As_ NNN complex center. Our GF calculations (cf. Section 4.3) have provided support to this argument.

### 4.3. Green’s Function Calculations

Accurate theoretical calculations are extremely valuable for providing confirmation to $N_{As}$, $N_{As}$–$N_{As}\;$and $N_{As}$–Ga–${Sb}_{As}$ data. Thus, it is essential to analyze the experimental results on impurity vibrational modes of N_As_ and Sb_As_ by carefully assessing the impurity–host interactions. With Raman scattering and FTIR spectroscopy the impurity modes of isolated defects in III–V compounds are extensively studied [75,76]. For the closest mass ^26^${Mg}_{Ga}\left(a^{-}\right)acceptor,\;$^27^${Al}_{Ga}\left(i\right)$ isoelectronic and ^28^${Si}_{Ga}\left(d^{+}\right)$ donors occupying the Ga site in GaAs revealed interesting results of LVMs. With respect to ${Al}_{Ga}\left(i\right)$, the ${Mg}_{Ga}\left(a^{-}\right)$ (${Si}_{Ga}\left(d^{+}\right)$) being a lighter (heavier) impurity atom indicated lower (higher) LVM frequency. This suggests a weaker ${Mg}_{Ga}^{-}$-As bonding than a stronger ${Si}_{Ga}^{+}$-As interaction. On the contrary, a closest mass $P_{As}(i)$ and ${Si}_{As}(a^{-})$ on the As site showed stronger bonds between ${Si}_{As}^{-}$-Ga than $P_{As}$-Ga bond. Similar behavior is noticed for $C_{As}^{-}$-Ga and $N_{As}$-Ga bonds in GaAs. Although these results are independent of the long-range Coulomb forces, our GF calculations strongly argue that the charged impurities in compound semiconductors affect only the short-range forces via redistribution of the electron-charge densities [55]. We have used these unique trends of force variations for predicting the LVMs of NN ($N_{As}$–${In}_{Ga}$) and NNN ${(N}_{As}$–Ga–${Sb}_{As})\;$pairs in GaAs. Next, we discuss the following cases:

(a) Single substitutional defect

**Figure 4:**
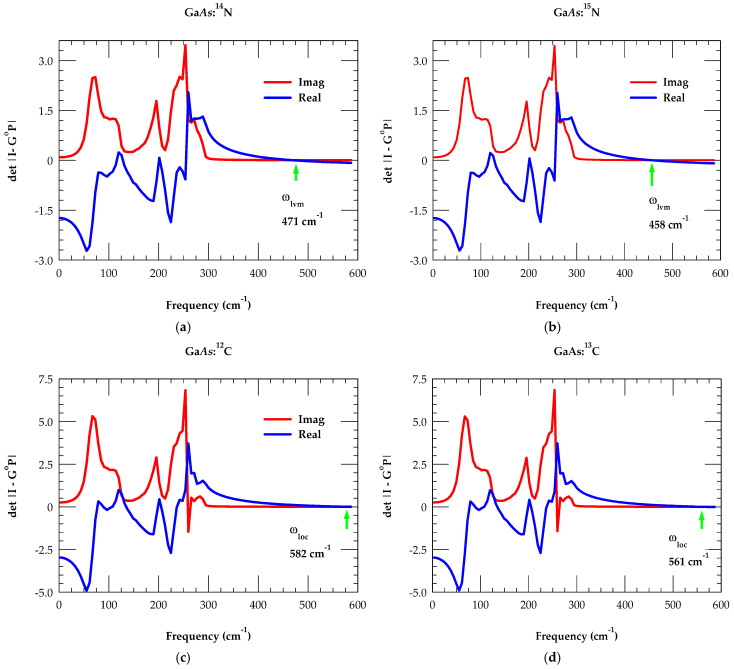
Calculated real (blue line) and imaginary (red line) parts of the det|I − G^o^P| (cf. Section 3.2.4 (a)) in the $F_{2}$ representation of the isolated N and C defects. The crossing of zero for the real det |I − G^o^P| provides local vibrational mode frequency (see: Table 2). (**a**) For GaAs: ^14^N at 471 cm^−1^; (**b**) for GaAs: ^15^N at 458 cm^−1^; (**c**) for GaAs: ^12^C at 582 cm^−1^; and (**d**) for GaAs: ^13^C at 561 cm^−1^ (see text).

Appropriate force constants in GF calculations are used (as discussed above) for isotopic $N_{As}$ (i) and $C_{As}^{-}$ acceptors in GaAs by calculating triply degenerate $F_{2}$ LVMs of ^14^N_As_, ^15^N_As_ and ^12^C_As_, ^13^C_As_ (see Figure 4a–d). The results are in good agreement with the existing FTIR data [25,26,27,28,29,30,31,32,33,34,35,36,37,38,39,40,41,42,43,44,45,46]. Similar calculations are also performed for isotopic N and C in GaSb.

(b) Nearest-neighbor pair

Adding a small amount of In in GaAs_1−x_N_x_ produces dilute In_y_Ga_1−y_As_1−x_N_x_ alloy. Replacing an ${In}_{1}$ atom with one of the four Ga-neighbors in Ga_4_N_As_ creates Ga_3_In_1_N_As_. This restructuring has produced an NN ${In}_{Ga}-N_{As}\;$pair defect of $C_{3v}$ symmetry. The involvement of heavier ${In}_{Ga}$ impurity atom causes splitting of $N_{As}$ triply degenerate $F_{2}$ LVM $(T_{d}$ symmetry) into a singlet $A_{1}$ and a doublet E. Such phonons are typically detected by using RSS [33] and/or IR [37] absorption spectroscopy.

(c) Next-nearest-neighbor complex

Again, the atomic configurations in the dilute $Ga{As}_{0.946}{Sb}_{0.032}N_{0.022}\;$alloy exhibits lower point group symmetry than$\;T_{d}\;$in Ga_4_N_As_. Replacing the As atom with Sb $\left({Sb}_{As}\right)\;$at the NNN site can form a complex center $N_{As}$–Ga–${Sb}_{As}$ of $C_{s}$ symmetry. However, substituting the As atom with N$\;\left(N_{As}\right)$ creates a $N_{As}$–Ga–$N_{As}$ pair defect of $C_{2v}$ symmetry. Experimental observation of their LVMs by RSS and FTIR can certainly offer information of restructuring, especially their bonding with $N_{As}$ after RTA.

In Table 2 we have reported the results of our systematic GF calculations of the LVMs and compared them with the existing experimental/theoretical data for isolated $T_{d}$, NN $C_{3v}$, and NNN $C_{2v}$/$C_{s}\;$pair defects.

The LVM of N isotopes in GaAs_1−x_N_x_, has provided vital information about the local atomic environment, i.e., N occupying a substitutional site or forming defect complexes. Measurements, in GaAs_1−x_N_x_ for x ⟶ 0, have exhibited a sharp high frequency mode of an isolated ^14^N_As_ defect near ~470–472 cm^−1^ [37]. Second-order phonon structure of the substitutional ^14^N_As_ mode has also been detected near 936 cm^−1^. Replacing ^14^N_As_ with a heavier ^15^N_As_ isotope has shifted the mode frequency to a lower value [37] in excellent agreement with our simulations (see Table 2). Based on a harmonic oscillator model, the LVM frequency of isotopic mass is inversely proportional to the square root of the mass of a vibrating atom. One can predict the local mode of a lighter isotope from the heavier one using the ratio of their masses $\frac{\omega_{LVM}^{N^{14}}}{\omega_{LVM}^{N^{15}}}=\sqrt{\frac{M_{15}}{M_{14}}}\;.$ Given the masses of ^14^N and ^15^N, the frequency of heavier isotopes (~455 cm^−1^) is expected to be approximately 96.6% of the lighter isotopic mass frequency 471 cm^−1^. Our calculated result for the heavier ^15^N isotope ~458 cm^−1^ agrees reasonably well with the above criteria. In GaSb, the calculated GF result of LVM for ^14^N_Sb_ (^15^N_Sb_) at ∼440 cm^−1^ (∼427 cm^−1^) (see Table 2) also satisfies the above condition. Although no experimental measurements are available for the N-isotopic defects in GaSb_1−x_N_x_, our GF result for ^14^N_Sb_ in GaSb is, however, slightly higher than that of the DFT value [44].

**Figure 5:**
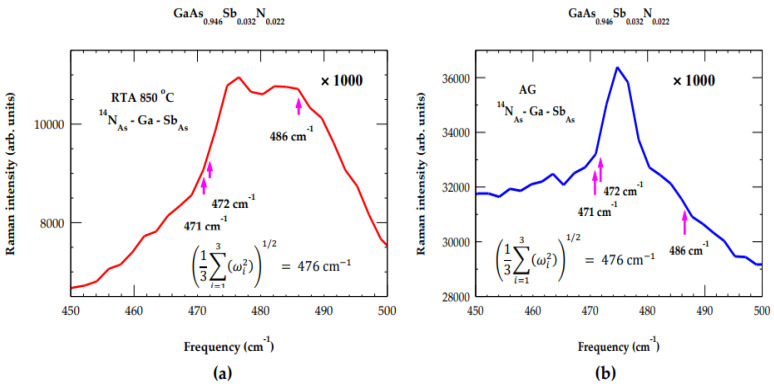
Amplified (×1000) Raman spectra of $Ga{As}_{0.946}{Sb}_{0.032}N_{0.022}/GaAs\;$ sample in the broad band 450–500 cm^−1^ spectral region for (**a**) RTA 850 °C and (**b**) AG. Calculated GF results of LVMs for the N_As_-Ga-Sb_As_ NNN complex center are shown using violet color arrows.

Unlike In_y_Ga_1−y_As_1−x_N_x_ where Raman [33,34] and IR [37] studies provided LVMs of isolated N_As_ (T_d_ symmetry) and NN In_Ga_-N_As_ pair (C_3v_ symmetry) defects, we did not find such measurements on as-grown and RTA GaAs_1−x−y_Sb_y_N_x_/GaAs (001) samples. The interpretation of Raman scattering spectra in $Ga{As}_{0.946}{Sb}_{0.032}N_{0.022}$ samples between the broad band 450–500 cm^−1^ region (see Figure 3) is much more complex. This intricacy is attributed to the breaking of translational symmetry and the involvement of either Ga-N, N-N, and/or other N-rich local configurations. Phonon features of different configurations could become more distinct due to strong Sb-N competition during the growth process. To visualize impurity modes more closely in $Ga{As}_{0.946}{Sb}_{0.032}N_{0.022}\;$samples (Figure 5a,b), we have amplified the Raman scattering results in the broad band region. This allows for the distinction between the highly disordered AG state and the improved crystalline structure of the RTA sample. The GF results of LVMs for the N_As_-Ga-Sb_As_ NNN complex center (C_s_ symmetry) are shown (see Table 2) by violet color arrows.

By applying a simple perturbation argument ω = ($\frac{1}{3}$Σ_i=1–3_($\omega_{i}^{2}$)^1/2^) [75,76] (see inset of Figure 5a,b) and using the fact that the three impurity modes at 471, 472 and 486 cm^−1^ of C_s_ symmetry are non-degenerate leads to ω = 476.3 cm^−1^. This phonon value is close to the observed frequency of the N_As_ (T_d_) local mode. Obviously, our analysis provides further support to the proposition for the formation of a N_As_-Ga-Sb_As_ NNN complex center. Based on spectroscopic behavior, it is often challenging to distinguish the impurity mode of Sb_As_ in GaAs due to heavier Sb-mass.

**Figure 6:**
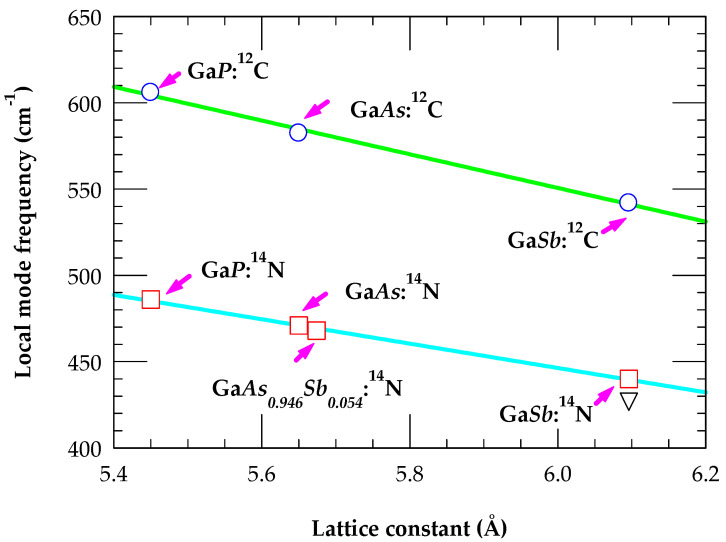
Best fit results (sky blue color line) of Green’s function calculation of F_2_ localized vibrational modes (LVMs) (cm^−1^) for isoelectronic defect ^14^N_As_ (open red color square, ours; inverted black color triangle, ref. [44]) as a function of lattice constant *a* (Å) for GaP, GaAs, GaAsSb, and GaSb. For GaAsSb, the value of relaxed *a* (Å) is deduced from X-ray measurements. Results of best fit calculations (green color line) of LVMs using GF method (open blue color circle) for ^12^C_As_ $\left(a^{-}\right)\;$ are also displayed for GaP, GaAs and GaSb.

In III–V (GaP, GaAs, GaAsSb and GaSb) compounds, we have also displayed (see Figure 6) our GF results of the LVM frequencies for the closest mass ^12^C_V_ ($a^{-}$) and ^14^N_V_ (i) as a function of lattice constant *a* (Å). Relaxed *a* (Å) for GaAsSb is deduced from the X-ray data. In each material, Figure 6 has clearly provided two valuable pieces of information about these defects: (a) the III-^12^C_V_ ($a^{-}$) bond is significantly stronger than the III-^14^N_V_ (i) bond, and (b) for each type of defect, the LVM frequency decreases linearly with the increase of *a* (Å).

In the dilute In_y_Ga_1−y_As_1−x_N_x_ alloy with low composition limits (i.e., x and y ⟶ 0), one expects the In atom to effectively substitute for the Ga site (${In}_{Ga}$). Strong affinity between $N_{As}$ and ${In}_{Ga}$ can trigger the formation of an NN ${In}_{Ga}$–$N_{As}\;$pair defect with lower $C_{3v}$ symmetry. As the pair is involved with a heavier ${In}_{Ga}$ atom, the triply degenerate $F_{2}$ mode of $N_{As}$ can split into a singlet $A_{1}$ and a doublet E. For the ${In}_{Ga}–N_{As}$ center, our GF calculation with appropriate values of change in force constants u, t and $F_{12}$ (see Section 3.2.4 (b)) in $\overset{⃡}{P}(\omega )$ has provided impurity mode frequencies near 465 cm^−1^ and 491 cm^−1^ (see Table 2) in good agreement with the experimental data [33,37].

In$\;Ga{As}_{0.946}{Sb}_{0.032}N_{0.022}$, one would expect the involvement of $N_{As}$ with$\;{Sb}_{As}$ to create an NNN center $N_{As}$-Ga-${Sb}_{As}.\;$Association of a ${Sb}_{As}\;$atom with $N_{As}$ and using appropriate values of change in force constants (see Section 3.2.4 (c)), our GF calculation provided splitting of $N_{As}$ LVM near ~476 cm^−1^ into three optically active non-degenerate $A_{1}$, $B_{1}$, and $B_{2}$ modes (see Table 2). Interestingly all these lines are falling in the broad band region of 450–500 cm^−1^, corroborating the perturbation model.

## 5. Conclusions

Systematic experimental and theoretical studies are essential for comprehending the profound alterations that N induces in the structural, optical, and impurity vibrational characteristics of the conventional III–V binary and ternary alloys. At low concentrations, N atoms in III–V-Ns are substitutional and preferentially occupy the group-V lattice sites. They can move to more energetically favorable locations at higher N concentrations due to the high miscibility gap and high electronegativity. Amongst the possible N-based quaternary alloys, GaAs_1−x−y_Sb_y_N_x_ is a promising material that can be grown lattice matched to GaAs. The incorporation of Sb and N in GaAs allows narrow band gaps 1.0–1.3 eV which are well suited for high-efficiency MJSCs and NIR PDs and LDs operating in the 1.31–1.55 μm range. Despite the significant efforts made in utilizing GaAs_1−x−y_Sb_y_N_x_ alloys in optoelectronic devices, the growth of high-quality layers on GaAs is significantly challenged primarily by the narrow low-temperature growth window and inherent difficulties of incorporating N into Sb-containing compounds. 

Local mode spectroscopy offers microscopic fingerprints for identifying N-related defects and clusters in III–V-N alloys and classifying the complex centers involving other atoms in quaternary materials. On the AG and annealed $Ga{As}_{0.946}{Sb}_{0.032}N_{0.022}$/GaAs (001) sample, we have performed Raman measurements to characterize not only the dynamical characteristics, but also its structural quality. The presence of N_As_ and ${Sb}_{As}$ atoms introduce distinct changes in the electronic and vibrational structures which can impact on the performance of different electronic devices. From the electronic structure standpoint, earlier measurements in GaAs_1−x−y_Sb_y_N_x_ [77] by deep-level-transient spectroscopy have detected two types of hole traps: (a) a shallow N-related $N_{As}$ defect state E_a_ ~0.10–0.12 eV and (b) an anti-site ${As}_{Ga}$ (${Sb}_{Ga}$)-related mid-gap state with E_a_ ~0.42–0.43 eV (~0.43 eV). Evidence for the change of ${As}_{Ga}$ from a deep-level to a shallow-level state has been speculated by the formation of NN ${As}_{Ga}$–$N_{As}$ pairs [77]. Our Raman scattering study has revealed several vibrational features at ω lower (<) and higher (>) than the maximum phonon frequencies of GaAs. Two vibrational modes $\omega_{LO(\Gamma )}^{GaAs}$ and $\omega_{TO(\Gamma )}^{GaAs}$ appearing at 293 cm^−1^ and 276 cm^−1^ exhibited almost identical frequencies with bulk GaAs [70]. This suggested that in the $Ga{As}_{0.946}{Sb}_{0.032}N_{0.022}\;$sample, the vibrational properties are dominated by strong long-range Ga-As bond networks rather than the impurity atoms. Observation of$\;\omega_{TO(\Gamma )}^{GaAs}\;$mode is usually caused by the breakdown of the standard Raman selection rule due to disordering. The presence of Sb in the sample has caused broadening of the high frequency triply degenerate N_As_ LVM near ~476 cm^−1^. We believe that the large FWHM of N_As_ mode is instigated by ${Sb}_{As}$ and not by an anti-site ${Sb}_{Ga}$ defect. The involvement of N_As_ with ${Sb}_{As}$ can create a complex defect center $N_{As}$-Ga-${Sb}_{As}$ of $C_{s}$ symmetry. We have calculated the LVMs of this impurity center by using GF methodology and considering $\overset{⃡}{P}(\omega )$ with appropriate force constant changes around the NNN sites. Using group theoretic arguments, the simulated results have revealed the splitting of the triply degenerate N_As_ mode into three optically active $A_{1}$, $B_{1}$, and $B_{2}$ phonon lines which fall in the broad band region of 450–500 cm^−1^. The analysis has provided support to our proposition about the formation of the N_As_-Ga-Sb_As_ complex center.

## Figures and Tables

**Table 1:** Rigid-ion parameters for the zb GaAs and GaSb materials.

Parameters ^a^	GaAs	GaSb
A	−0.4071	−0.35
B	−0.166	−0.262
C_1_	−0.0177	−0.0190
C_2_	−0.0461	−0.0280
D_1_	0.0248	−0.0668
D_2_	−0.1233	0.023
E_1_	0.0912	0.07
E_2_	0.0834	−0.12
F_1_	−0.1172	0.13
F_2_	0.2008	−0.119
Z_eff_	0.658	0.4840

^a^ Ref. [59].

**Table 2:** Rigid-ion model Green’s function calculations for localized vibrational mode frequencies (in cm^−1^) associated with different N configurations in zb GaAs and GaSb.

Symmetry	Configuration	LVMs in GaAs		Configuration	LVMs in GaSb	
		Our	Others ^a^		Our	Others ^b^
$T_{d}$	^14^ $N_{As}$ ^15^ $N_{As}$	471 458	470, 471 458	^14^ $N_{Sb}$ ^15^ $N_{Sb}$	440 427	427.6
$C_{3v}$	^14^$N_{As}$-In_Ga_ ^15^$N_{As}$-In_Ga_	491, 465 476, 451	488, 459; 488, 468			
$C_{2v}$	^14^$N_{As}$-Ga-^14^$N_{As}$ ^15^$N_{As}$-Ga-^15^$N_{As}$	501 480 476 459 454 429 487 466 462 446 440 417		^14^$N_{Sb}$-Ga-^14^$N_{Sb}$ ^15^$N_{Sb}$-Ga-^15^$N_{Sb}$	473 465 455 440 429 416 460 449 440 425 415 403	449.1 445.9 428.0 415.4 339.7 324.2
$C_{s}$	^14^$N_{As}$-Ga-${Sb}_{As}$ ^15^$N_{As}$-Ga-${Sb}_{As}$	486 472 471 472 458 457				

^a^ Refs. [25,26,27,28,29,30] ^b^ Ref. [44].

## Data Availability

The original contributions presented in this study are included in the article. Further inquiries can be directed to the corresponding author.
